# Impact of Anti‐obesity Medication Initiation and Duration on Weight Loss

**DOI:** 10.1002/osp4.70069

**Published:** 2025-03-21

**Authors:** M. Rami Bailony, Paulina Garzon Espinoza, Harun Yüksel, Imran M. Kyeso, Roxana Khalili, Sheryl Haller

**Affiliations:** ^1^ Enara Health Inc. San Mateo California US; ^2^ San Francisco State University San Francisco California US; ^3^ University of California San Diego California US; ^4^ University of California Merced California US

**Keywords:** anti‐obesity, drug treatment, obesity treatment, outcomes, weight loss

## Abstract

**Introduction:**

Real‐world studies of anti‐obesity medication (AOM) use have shown lower adherence and persistence than clinical trials; however, the impact of this reduced adherence in real‐world settings remains largely unexplored. This study aimed to evaluate the effects of AOM use, timing of initiation, and duration on 18‐month weight loss outcomes in comprehensive obesity care practice, offering critical insights into the role of adherence in optimizing treatment efficacy.

**Methods:**

This retrospective cohort study assessed the electronic health records of adults with a body mass index (BMI) ≥ 30 kg/m^2^ enrolled in a digital obesity program for ≥ 18 months. Participants were categorized by AOM use, initiation timing (early vs. delayed), and duration (short vs. long).

**Results:**

This study of 1282 participants showed that AOM users had greater weight reduction than non‐users. Long‐ and short‐duration AOM users experienced significantly more weight loss than short‐duration users, with no difference between early and delayed starters. Second‐generation semaglutide users were more likely to reach the 20% weight loss milestone, especially with longer use.

**Conclusion:**

Long‐term AOM use significantly improved weight loss in comprehensive obesity care. Furthermore, the success of non‐AOM users highlights the value of intensive behavioral programs, indicating the need for personalized treatment to optimize cost‐effectiveness and outcomes.

## Graphical abstract

This retrospective study of 1282 patients found that long‐term AOM usage contributed to significant weight loss (14.70%–17.34%) compared to short term AOM use (9.25%–12.56%). The study observes that non‐AOM users within a structured obesity care program can also achieve notable weight loss (9.99%–12.77%), highlighting the potential effectiveness of intensive behavioral therapy without pharmacotherapy.

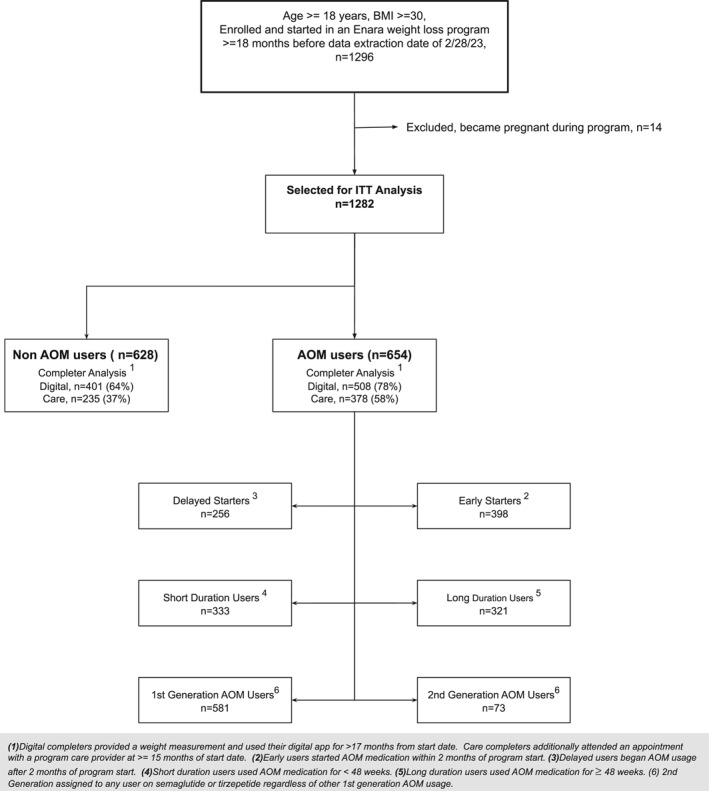

## 1 Introduction

The advent of second‐generation anti‐obesity medications (AOMs), notably semaglutide and tirazepatide, represents a significant advancement in the treatment of obesity [1, 2]. This development is at a critical juncture in addressing obesity as a public health crisis against valid concerns regarding the cost‐effectiveness of new therapies [3].

In real‐world settings, AOMs are prescribed to a broader population than those typically included in randomized clinical trials (RCTs), such as individuals with recent weight changes or psychiatric conditions [4, 5, 6]. Furthermore, real‐world practice varies significantly in the intensity, capabilities, and adherence of the concomitant, behavioral, and lifestyle programs compared to those in RCTs [7]. This highlights the need to understand the performance of AOMs under various conditions. The following key research questions have emerged: What is the optimal timing for initiating AOMs in the context of comprehensive obesity care [8, 9]? What are the effects of medication duration on AOM adherence? How does lifestyle, behavioral care delivery, and adoption affect AOM efficacy and maintenance [10]?

In real‐world studies, adherence to first‐ and second‐generation AOMs at 12 months was reported to range from 2% to 22% [11, 12] and 38%–60%, respectively [13, 14, 15, 16]. In comparative studies, the absolute treatment difference between AOM and non‐AOM groups has underperformed that of RCTs [17, 18, 19, 20, 21]. In contrast, in non‐comparative studies, second‐ and first‐generation AOMs have been found to produce weight loss effects similar to those reported in RCTs for completers [5, 6, 15, 16, 22, 23]; however, upon adjusting for real‐world adherence with intention‐to‐treat analyses, the mean weight loss has been reported to be 35%–50% lower than that in RCTs [14, 15, 24, 25, 26]. Real‐world studies often have variable definitions of “completers,” varying between digital evidence (e.g., medication claims, refill logs, or mobile tracking) and care cutoffs (e.g., medical follow‐ups or session completion) [7]. This variability compromises generalizability, as it overlooks crucial differences in adherence to medication, lifestyle, and behavioral interventions, which are essential for informing care effectiveness and a broader health policy. Furthermore, the prevalent focus on analyses of completers rather than an intention‐to‐treat approach limits the ability to compare these findings directly with RCTs, which typically assess the effectiveness of treatment initiation.

This study aimed to address these questions by examining the use of AOMs within a comprehensive lifestyle obesity care clinic framework. We assessed the impact of medication use, timing, and duration. Our study addresses a critical gap in the real‐world evidence by examining the differential impact of long‐term versus short‐term adherence to first‐ and second‐generation AOMs benchmarked against outcomes in a non‐medication intervention group. While previous studies have explored weight loss outcomes and adherence in 2nd generation medication users, ours is the first study to integrate a lifestyle‐focused comparator, providing a nuanced perspective on the incremental value of sustained AOM adherence or the true impact of non‐adherence in an obesity care model that includes comprehensive lifestyle and behavioral interventions.

## 2 Methods

### 2.1 Study Overview

This retrospective cohort study analyzed de‐identified electronic health record data from participants enrolled in a digital obesity program, extracted in February 2023. The study adhered to the Strengthening the Reporting of Observational Studies in Epidemiology (STROBE) guidelines and received exemption approval (ID: 10299‐MRBailony) from the Sterling Institutional Review Board on September 1, 2022, in accordance with Health and Human Services regulations 45 CFR 46.101(b) [4].

### 2.2 Participant Selection

This study included individuals aged ≥ 18 years with a body mass index (BMI) ≥ 30 kg/m^2^ who were enrolled in the Enara program for ≥ 18 months from the data pull date. Participants who became pregnant during the study period were excluded (*n* = 14). Subgroups were assigned according to AOM use (users vs. non‐users), timing of AOM initiation (early vs. delayed), treatment duration (short‐term vs. long‐term), and AOM generation (first vs. second).

AOM users were defined as those taking AOMs based on electronic health records or Surescripts prescription data for ≥ 60 days. All other participants were assigned to the non‐AOM group. A 60‐day cutoff was used to accurately identify participants who were prescribed AOMs but did not initiate treatment. Surescript data corroborated this classification. AOM initiation status was assigned based on the date of the first AOM prescription. The duration of AOM treatment was determined from the initiation date of the first AOM prescription to the discontinuation date of all AOM prescriptions or at the end of the 72‐week study period, with adjustments made by subtracting any gap days in therapy. Early starters were defined as those initiating an AOM within 2 months of the start of the program, whereas delayed starters initiated medication thereafter. Short‐duration AOM users were defined as those who received one or more AOM for < 48 weeks. Long‐term AOM users were defined as participants who received AOMs for ≥ 48 weeks. Participants treated with semaglutide or tirzepatide were classified as second‐generation AOM users, irrespective of the use of other primary or adjunctive AOMs. Those receiving all other anti‐obesity medication treatments were categorized as first‐generation AOM users.

### 2.3 Intervention

Enara Health is an obesity care platform that extends from primary care and cardiology clinics to virtual obesity and cardiometabolic departments. Participants were initially evaluated by a physician or physician assistant who determined the severity of obesity and readiness to change. The participants subsequently underwent a full metabolic work‐up and examination. Participants then attended routine telehealth appointments with nutritional or exercise specialists and were supported by a mobile app for education, encouragement, and tracking. Participants could choose between an intense, rapid dietary program (daily caloric intake of 800–1200 cal) or a gradual program that did not encourage calorie counting. Both programs encouraged the consumption of whole, unprocessed, and low‐glycemic foods. Exercise targets were individualized for each participant; however, a minimum of 150 min/week was set for all participants. Many participants utilized activity trackers such as Fitbit or Apple Watch to monitor weekly exercise.

Where appropriate, the participants were prescribed AOMs. All patients were started on the lowest medication dosage, and dosages were adjusted individually as needed to achieve the desired outcomes. The individual participants and their physicians chose the medications. Some participants may have been prescribed multiple medications for obesity.

### 2.4 Data Collection

Enara collected demographic and clinical data during initial clinic visits. All participants had their height, body weight, blood pressure, pulse, and body composition measured at the initial appointment to establish a baseline, which was then re‐checked in the clinic at in‐person appointments. Body weight was also collected daily from the participant's home scale, which connects directly to Enara Health's provider dashboard. Medical providers recorded medical compliance on a monthly or quarterly basis.

### 2.5 Study Endpoints

The first primary endpoint was the percentage weight change from baseline at 72 weeks. The second primary endpoint was the proportion of patients who lost at least 5%, 10%, 15%, or 20% of their baseline body weight.

### 2.6 Statistical Analysis

For the primary analysis, an intention‐to‐treat last observation carried forward (ITT LOCF) analysis was performed. If missing, the imputed data were obtained from the last measured observation. Thereafter, two complete analyses were performed to evaluate the impact of engagement on the outcomes. Digital completers were users who logged their weights weekly and checked the Enara App monthly ± 1 month from the cohort end date. Care completers were those who additionally had one medical or behavioral health appointment within 3 months of the cohort end date.

Statistical analyses were conducted using Python 3.10 with the SciPy library. Histograms and Kolmogorov–Smirnov tests were used to assess the data distribution. Statistical significance was set at a threshold of *p* < 0.05. Independent‐sample t‐tests were used for two‐group comparisons involving continuous data. In cases of low patient counts or when the data did not follow a normal distribution in the subgroup comparisons, the Mann–Whitney *U* test was applied. Three or more group comparisons were performed using the one‐way ANOVA, followed by Tukey's Honestly Significant Difference post hoc test for normally distributed data. Non‐parametric multiple‐group comparisons were conducted using the Kruskal–Wallis test, with post hoc Mann–Whitney U tests incorporating the Bonferroni correction. Nominal data comparisons in the two‐group analyses were performed using the chi‐square test. The chi‐square test with Bonferroni correction as a post hoc test was employed for three or more group comparisons involving nominal data.

## 3 Results

### 3.1 Baseline Characteristics

**FIGURE 1:**
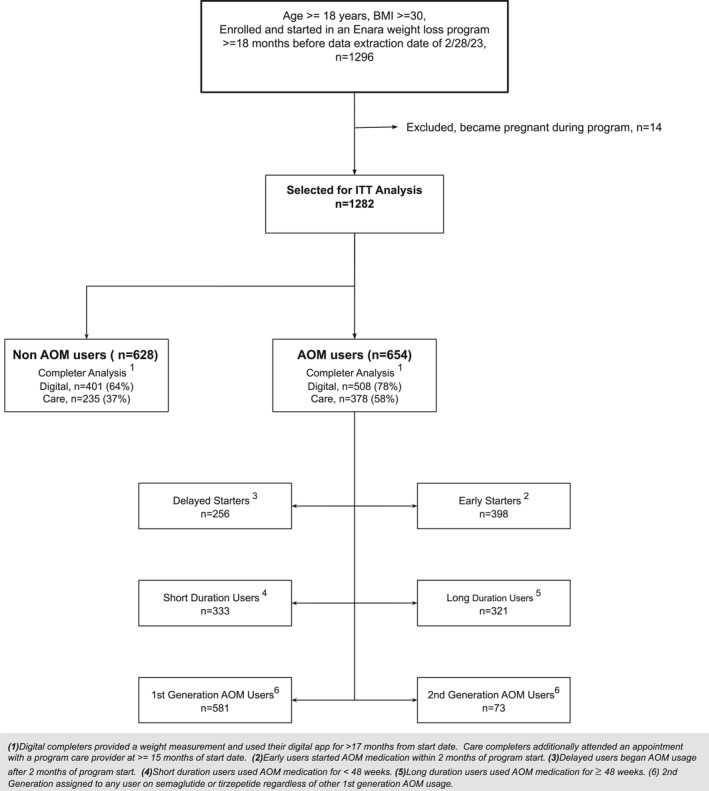
Participant flow diagram.

A total of 1282 participants (628 non‐AOM and 654 AOM users) met the ITT criteria. Of the AOM users, 78% were digital completers compared with 64% of non‐AOM users. In addition, 58% of AOM users were care completers compared with 37% of non‐AOM users. Among the AOM users, 61% were early starters, and 42% were long‐duration users (Figure 1). In total, 11.3% of AOM users were on a second‐generation AOM, all of which were semaglutide with no documented tirzepatide use (Table 1, Figure 1).

**TABLE 1:** Participants' baseline characteristics.

Baseline characteristics	Non‐AOM users *n* = 628	AOM users *n* = 654
Age (yr.)	46.9 ± 13.7	46.4 ± 12.3
Baseline weight (kg)	100.5 ± 20.5*	105.5 ± 22.4*
BMI (kg/m2)	36.3 ± 5.8*	38.0 ± 6.6*
Gender (male). *n* (%)	196 (31)*	168 [26]*
Gender (female). *n* (%)	432 (69)*	486 (74)*
hbA1C (%)	5.7 ± 0.8	5.7 ± 1.1
Glucose (mg/dL)	100.3 ± 25.8	102.7 ± 32.7
Insulin	13.4 ± 10*	15.6 ± 13.6*
Total cholesterol (mg/dL)	194 ± 47.9	193.5 ± 41.8
Triglycerides (mg/dL)	126.9 ± 80.1	129.4 ± 79.2
LDL‐cholesterol (mg/dL)	116.6 ± 34	116.1 ± 35.6
VLDL‐cholesterol (mg/dL)	23.5 ± 13.3	23.1 ± 16.7
HDL‐cholesterol (mg/dL)	52.5 ± 14.1	53.6 ± 14.5
HsCRP (mg/dL)	3.7 ± 3	4 ± 3.4
AOM start date ‐ program start date (mean)	—	75.8 days
AOM duration (mean)	—	271.7 days
Participant usage by medication (%)		
Bupropion		11.1%
Bupropion/Naltrexone		6.4%
Naltrexone		5.2%
Phentremine		50.7%
Phentermine/Topiramate		7.9%
Topiramate		40.8%
Liraglutide		21.4%
Semaglutide		11.2%
Tirzepatide		0.0%

*Note:* Values shown are *n* (%) or means ± standard deviation. *Categories differ significantly from each other at *p* < 0.05.

Abbreviations: HbA1c, Hemoglobin A1c; HDL, High‐Density Lipoprotein; HsCRP, High Sensitivity C‐Reactive Protein; LDL, Low‐Density Lipoprotein; VLDL, Very Low‐Density Lipoprotein.

Table 1 shows the baseline characteristics of the participants. AOM users had a significantly higher percentage of females (74 vs. 69, *p* = 003), baseline BMI (38.0 vs. 36.3 kg/m^2^, *p* < 0.001), weight (105.5 vs. 100.5 kg, *p* < 0.001), and fasting insulin (15.6 vs. 13.4 µIU/mL, *p* = 0.02).

### 3.2 AOM Use versus Non‐AOM Use

Participants using AOM medication experienced a significantly larger average reduction in their initial weight percentage compared to those not using medication across all analyses: in the ITT group, −11.75% versus −9.99% (*p* < 0.001); in digital completers, −12.67% versus −11.21% (*p* = 0.017); and in care completers, −14.42% versus −12.77% (*p* = 0.02). Additionally, AOM users showed a significantly higher percentage of individuals achieving weight loss milestones of 5%, 10%, 15%, and 20% by the end of the study in the ITT and digital completer groups; however, in the care completer group, significance was observed only for the 20% weight loss milestone. Further details are presented in Table 2.

**TABLE 2:** Changes in primary end points (AOM users ‐ non‐users).

	Non‐AOM users (n)	AOM users (*n*)	Difference between groups (95% CI)	*p* value
Change in weight (%)				
ITT analysis	−9.99% (628)	−11.75% (654)	**−1.76, [0.813, 2.693]**	**< 0.001**
Digital completers	−11.21% (401)	−12.67% (508)	**−1.46, [0.259, 2.673]**	**0.02**
Care completers	−12.77% (235)	−14.42% (378)	**−1.65, [0.246, 3.058]**	**0.02**
≥ 5% of body weight				
ITT analysis	71.0%	77.5%	**6.5, [1.7, 11.3]**	**0.0093**
Digital completers	75.1%	79.7%	4.6, [−0.9, 10.1]	0.1106
Care completers	83.4%	86.2%	2.8, [−3.1, 8.7]	0.3984
≥ 10% of body weight				
ITT analysis	45.3%	54.5%	**9.2, [3.7, 14.7]**	**0.0012**
Digital completers	52.9%	60.0%	**7.1, [0.6, 13.6]**	**0.0357**
Care completers	63.8%	69.8%	6.0, [−1.7, 13.7]	0.1452
≥ 15% of body weight				
ITT analysis	24.0%	33.3%	**9.3, [4.4, 14.2]**	**< 0.001**
Digital completers	29.9%	38.6%	**8.7, [2.5, 14.9]**	**0.0080**
Care completers	37.9%	45.2%	7.3, [−0.7, 15.3]	0.0873
≥ 20% of body weight (%)				
ITT analysis	11.0%	18.7%	**7.7, [3.8, 11.6]**	**< 0.001**
Digital completers	15.2%	22.2%	**7.0, [2.0, 12.0]**	**0.0096**
Care completers	19.1%	27.0%	**7.9, [1.2, 14.6]**	**0.0347**

*Note:* All changes are from baseline to 18 months. Values shown are means ± standard deviation or % of weight reduction target achieved. Bold Categories differ significantly from each other at *p* < 0.05.

### 3.3 Early versus Delayed Starters

There were no significant differences in the mean reduction in baseline weight percentage or categorical weight loss outcomes between the early and delayed medication starters (Supporting Information S1: Table S1).

### 3.4 Short versus Long‐Duration Users

Long‐duration AOM users were notably older at the start (48.4 years compared to 44.5 years, *p* < 0.001) and had higher hemoglobin A1C (HbA1c; 5.8% compared to 5.6%, *p* = 0.18). However, they showed no other significant differences in BMI, sex, or other baseline cardiometabolic markers compared with those with short‐term AOM use (Supporting Information S1: Table S2).

Longer AOM use was associated with significantly larger reductions in the baseline weight across all analyses: 14.70% versus 9.58% in the ITT group (*p* < 0.001), 15.03% versus 10.42% among digital completers (*p* < 0.001), and 15.89% versus 12.56% among care completers (*p* < 0.001), as shown in Table 3.

**TABLE 3:** Changes in primary end points (long duration ‐ short duration).

	Short duration AOM users (*n*)	Long duration AOM users (n)	Difference between groups (95% CI)	*p* value
Change in weight (%)				
ITT analysis	−9.25% (333)	−14.34% (321)	**5.09, [3.782, 6.398]**	**< 0.001**
Digital completers	−10.04% (224)	−14.75% (284)	**4.71, [3.134, 6.289]**	**< 0.001**
Care completers	−12.26 (138)	−15.66% (240)	**3.40, [1.631, 5.175]**	**< 0.001**
≥ 5% of body weight				
ITT analysis	68.8%	86.6%	**17.8, [11.6, 24.0]**	**< 0.001**
Digital completers	70.1%	87.3%	**17.2, [10.1, 24.3]**	**0.0020**
Care completers	79.7%	90.0%	**10.3, [2.6, 18.0]**	**0.0051**
≥ 10% of body weight				
ITT analysis	40.5%	69.5%	**29.0, [21.7, 36.3]**	**< 0.001**
Digital completers	45.5%	71.5%	**26.0, [17.6, 34.4]**	**< 0.001**
Care completers	58.7%	76.2%	**17.5, [7.7, 27.3]**	**< 0.001**
≥ 15% of body weight				
ITT analysis	20.7%	46.4%	**25.7, [18.7, 32.7]**	**< 0.001**
Digital completers	25.4%	48.9%	**23.5, [15.4, 31.6]**	**< 0.001**
Care completers	33.3%	52.1%	**18.8, [8.7, 28.9]**	**0.0062**
≥ 20% of body weight (%)				
ITT analysis	11.4%	26.2%	**14.8, [8.9, 20.7]**	**< 0.001**
Digital completers	15.6%	27.5%	**11.9, [4.9, 18.9]**	**< 0.001**
Care completers	21.7%	30.0%	**8.3, [‐0.7, 17.3]**	**0.0031**

*Note:* All changes are from baseline to 18 months. Values shown are means ± standard deviation or % of weight reduction target achieved. Bold Categories differ significantly from each other at *p* < 0.05.

Furthermore, longer AOM use was correlated with a significantly higher proportion of participants achieving weight loss milestones of 5%, 10%, 15%, and 20% across all evaluated groups (see Table 3).

The long‐ and short‐duration cohorts were subsequently assessed compared to non‐AOM users. Long‐duration AOM use was associated with significantly improved mean and categorical weight loss in all analyses (Supporting Information S1: Table S3). There were no significant differences between short‐term AOM and non‐AOM users in the mean reduction in baseline weight percentage or categorical weight loss outcomes (Supporting Information S1: Table S4).

### 3.5 First versus Second‐Generation AOMs

**FIGURE 2:**
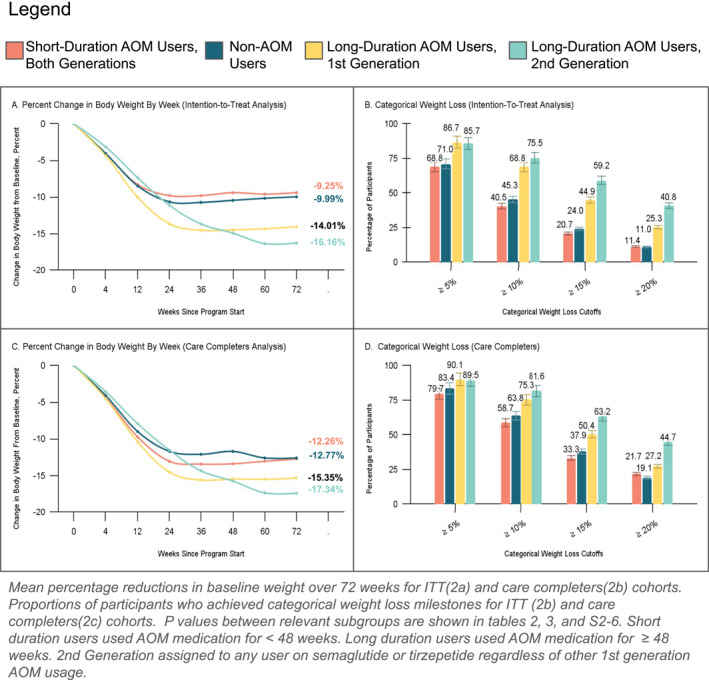
Weekly percentage change in body weight and categorical weight loss milestones.

Across all analyses, there was no significant difference in the baseline weight reduction between second‐generation and first‐generation AOM users: ITT (−13.58% vs. −11.58%, *p* = 0.06), digital completers (−14.02% vs. −12.49%, *p* = 0.23), and care completers (−15.33% vs. −14.28%, *p* = 0.42). Among long‐duration medication users, superior but non‐significant trends between second and first‐generation AOMs were also observed across all groups: ITT (−16.16% vs. −14.01%, *p* = 0.12), digital completers (−16.21% vs. −14.48%, *p* = 0.23), and care completers (−17.34% vs. −15.35%, *p* = 0.17) (Supporting Information S1: Tables S5 and S6 in and Figures 2a and 2b).

A significantly larger proportion of second‐generation AOM users achieved 20% weight loss in the ITT analysis (30.14% vs. 18.09%, *p* = 0.02). Among users with a longer AOM duration, second‐generation AOM users had a higher percentage of participants achieving 20% weight loss than their first‐generation counterparts across all analyses: ITT (40.8% vs. 25.5%, *p* = 0.018), digital completers (40.9% vs. 25%, *p* = 0.047), and care completers (44.7% vs. 27.2%, *p* = 0.049) (Supporting Information S1: Table S6 in and Figure 2d).

## 4 DISCUSSION

This retrospective cohort study critically evaluated the real‐world efficacy of AOMs using a comprehensive obesity care clinical model. Furthermore, these outcomes were compared with a cohort of non‐AOM users, offering novel insights into the role of AOMs in real‐world practice.

Real‐world studies from academic medical centers reported an average weight loss of 10.2%–10.5% over 24 months in patients who started and continued a first‐generation AOM [5, 6, 22]. These studies examined patients who started medication at onset and completed a follow‐up; therefore, these studies were the most comparable to our care completers analysis of long‐term first‐generation AOM users, who saw a 15.35% loss. The care follow‐up rate of AOM users in the present study was 49.9%, which was higher than the 17%–27% reported previously [5, 6, 22].

Early studies on second‐generation AOMs reported an average weight loss ranging from 6.0% in ITT analyses to 13.2% in care completer analyses at 52 weeks [15, 23, 26]. Adherence or completion at 12 months has been reported as 37%–50% at 12 months for semaglutide [13, 14, 15, 16]. Comparatively, in our study, second‐generation AOM users, particularly those treated for more than 48 weeks, comprised 16.21% of the ITT group and 17.34% of the care completers. Second‐generation AOM users had a completion rate of 71%. Our results showed higher achievements in weight loss milestones, with 90.0%, 76.2%, 52.1%, and 30% of AOM completers achieving 5%, 10%, 15%, and 20% weight loss, respectively, despite a second‐generation AOM use rate of only 11.2%. These outcomes are in agreement with those reported in Step I semaglutide trials [4].

The robust outcomes delivered in this clinical model without medication may explain these superior outcomes. Non‐AOM users achieved an average weight loss of 9.99% at 18 months on an ITT basis, whereas care users achieved a weight loss of 12.77%. In the few real‐world studies comparing AOM and non‐AOM groups, the treatment difference consistently underperformed that of RCTs [17–21]. In real‐world practice, individuals are not randomized to receive or not receive medication. Patients with a severe history of weight recidivism were more likely to opt for medication. The fact that the AOM users had significantly higher BMIs supports this finding. Additionally, patients who struggle with lifestyle and behavioral programs alone are more likely to start medication later. This finding highlights the strengths of this study. By dividing AOM usage into cohorts based on initiation and duration against the baseline, we could better determine the clinical impact and cost‐effectiveness. Subsequently, on an ITT basis, AOM usage for ≥ 48 weeks resulted in almost 50% more weight loss than that in non‐AOM users (14.70% vs. 9.99%) despite a higher BMI and cardiometabolic risk profile at baseline. While many studies have reported poor adherence, our study is the first to quantify the impact of AOM non‐adherence, showing that use for < 48 weeks may not produce additional health benefits compared to behavioral therapy in real‐world scenarios.

This study also underscores that significant weight loss can be achieved without relying solely on second‐generation AOMs. The observed weight loss in the non‐AOM ITT and completer groups indicated that many individuals can attain meaningful weight loss through intensive behavioral programs alone. The advent of second‐generation AOMs has shifted the discourse on lifestyle and behavioral therapy to how such programs or services can improve or augment the drug therapy response [8, 10, 27]. However, this shift has overlooked the intrinsic value of lifestyle and behavioral modification programs, which have demonstrated health benefits beyond weight loss. An equally important question is how AOMs can complement and sustain the changes brought about by lifestyle and behavioral interventions, thereby offering a more comprehensive and holistic approach to chronic diseases and obesity.

As AOM costs increase, our findings advocate for a more nuanced approach to the treatment of obesity. Recognizing the diversity in patient responses to different treatment modalities, it is imperative to avoid homogenizing the medical treatment for obesity. This strategy could lead to better treatment personalization and potential cost savings, particularly for individuals who succeed with first‐generation medications or non‐pharmacological methods. To support this differentiated approach, payors can implement payment modifiers based on weight loss outcomes or medication utilization, further incentivizing cost‐efficient treatment strategies. The economic implications of such a policy, which focuses on the right treatment for the right patient, should not be overlooked. Second, there is a need for strategies that focus on enhancing patient compliance and engagement, especially when medication is not used.

Despite these insights, our study had limitations inherent to its retrospective design, including potential bias and constraints in establishing causality. The number of participants taking second‐generation GLP‐1 was limited by the data pull date against the 18‐month ITT endpoint, underpowering the subgroup. Second, the comprehensive obesity care model makes deciphering and controlling for confounding variables difficult. Late medication initiation could be due to poor behavioral or lifestyle responses, biasing our cohort selection by excluding them from the non‐medication ITT group. Third, while we studied medication adherence and duration, we did not collect or analyze participants' adherence to the behavioral and nutritional components of the program, which may have a compounding impact on outcomes. For example, medication users were less likely to attend nutrition or exercise sessions than non‐medication users. Finally, our study methodology, which considers ITT and completion based on enrollment and ongoing participation in obesity care, stands apart from other studies that focused mainly on medication initiation and follow‐up. Although this approach may complicate direct comparisons, it underscores the need for a more holistic view of obesity treatment, advocating for research that explores the combined effects of multiple treatment modalities. This comprehensive approach is vital to enhance our understanding and implementation of effective obesity management strategies.

In summary, this study provided a comprehensive evaluation of obesity treatments, emphasizing the importance of exploring how various interventions interact and complement each other to foster effective and value‐driven care.

## Conflicts of Interest

Ms. Garzon, Mr. Kyeso, and Ms. Khalili are not compensated for this study and have no conflicts of interest. The remaining authors work for and/or have shareholder interests in Enara Health.

## Supporting information

Supporting Information S1

## Data Availability

De‐identified participant data and the data dictionary that underlies the results reported in this article can be made available for research collaboration upon request. Requests for research applications, data processing agreements, and data use approval forms should be sent to the corresponding authors.
