# Supplementary material for: Impact of Anti‐obesity Medication Initiation and Duration on Weight Loss
Source: Obes Sci Pract. 2025 Mar 21;11(2):e70069. doi: 10.1002/osp4.70069 (PMC11928677; doi:10.1002/osp4.70069)
Supplement: Supplementary file 1 — Supporting Information S1 [file OSP4-11-e70069-s001.docx]

**SUPPLEMENT1**

**The Impact of Anti-Obesity Medication Initiation and Duration on Weight Loss**

Bailony, MR; Espinoza, P; Yüksel, H; Kyeso, I; Khalili, R; Haller, S

**Contents**

- **Table S1** - Changes in Primary and Secondary Endpoints (Early - Delayed)
- **Table S2** - Long Duration vs Short Duration AOM Users Baseline Characteristics
- **Table S3** - Changes in Primary And Secondary Endpoints (Long Duration AOM- Non-AOM Group )
- **Table S4** - Changes in Primary And Secondary Endpoints (Short Duration AOM- Non-Medication Group )
- **Table S5**. Changes in Primary End Points (2nd generation - 1st Generation)
- **Table S6**. Changes in Primary End Points (2nd Generation Long - 1st Generation Long)

| **Table S1. Changes in Primary And Secondary End Points (Early - Delayed)** | | | | |
| --- | --- | --- | --- | --- |
|  | **Delayed**  **(n )** | **Early**  **(n)** | **Difference Between Groups (95% CI)** | **P Value** |
| **Change in weight (%)** |  |  |  |  |
| ITT analysis | 11.11% (256) | 12.15% (398) | 1.0, [-0.64, 1.95] | 0.319 |
| Digital Completers | 12.20% (202) | 12.99% (306) | 0.8, [-0.86, 2,44] | 0.349 |
| Care Completers | 14.06% (151) | 14.66% (227) | 0.6, [-1.18, 2.36] | 0.510 |
| **≥ 5% of body weight** |  |  |  |  |
| ITT analysis | 75.0% | 79.1% | 4.1, [-2.5, 10.7] | 0.253 |
| Digital Completers | 78.7% | 80.4% | 1.7, [-5.5, 8.9] | 0.728 |
| Care Completers | 85.4% | 86.8% | 1.4, [-5.7, 8.5] | 0.824 |
| **≥ 10% of body weight** |  |  |  |  |
| ITT analysis | 51.2% | 57.0% | 5.8, [-2.0, 13.6] | 0.165 |
| Digital Completers | 57.9% | 61.4% | 3.5, [-5.2, 12.2] | 0.484 |
| Care Completers | 67.5% | 71.4% | 3.9, [-5.6, 13.4] | 0.498 |
| **≥ 15% of body weight** |  |  |  |  |
| ITT analysis | 29.7% | 35.7% | 6.0, [-1.3, 13.3] | 0.133 |
| Digital Completers | 34.7% | 41.2% | 6.5, [-2.1, 15.1] | 0.166 |
| Care Completers | 41.7% | 47.6% | 5.9, [-4.3, 16.1] | 0.310 |
| **≥ 20% of body weight (%)** |  |  |  |  |
| ITT analysis | 16.4% | 20.1% | 3.7, [-2.3, 9.7] | 0.280 |
| Digital Completers | 19.3% | 24.2% | 4.9, [-2.4, 12.2] | 0.236 |
| Care Completers | 24.5% | 28.6% | 4.1, [-4.9, 13.1] | 0.443 |
| All changes are from baseline to 18 months. Values shown are means ± standard deviation or % of weight reduction target achieved. Bold Categories differ significantly from each other at P < 0.05. | | | | |
|  |  |  |  |  |

| **Table S2. Long Duration vs Short Duration AOM Users Baseline Characteristics** | | |
| --- | --- | --- |
| **Baseline Characteristics** | **Short Duration AOM Users (n=333)** | **Long Duration AOM Users (n=321)** |
| Age (yr.) | 44.8 ± 12.1* | 48.4 ± 12* |
| Baseline weight (kg) | 101.9 ± 19 | 106.4 ± 23.7 |
| BMI (kg/m2) | 36.9 ± 5.9 | 38.3 ± 6.8 |
| Gender (male). n (%) | 53 ± 24 | 70 ± 25 |
| Gender (female). n (%) | 171 ± 76 | 214 ± 75 |
| hbA1C (%) | 5.6 ± 0.9* | 5.8 ± 1.2* |
| Glucose (mg/dL) | 100.3 ± 25.4 | 105.1 ± 38.4 |
| Insulin | 14.4 ± 12.1 | 16.6 ± 15 |
| Total cholesterol (mg/dL) | 192.3 ± 40.9 | 194.6 ± 42.6 |
| Triglycerides (mg/dL) | 123.8 ± 76.9 | 134.9 ± 81.2 |
| LDL-cholesterol (mg/dL) | 116.6 ± 35.8 | 115.6 ± 35.5 |
| HDL-cholesterol (mg/dL) | 52.7 ± 14.5 | 54.5 ± 14.5 |
| VLDL-cholesterol (mg/dL) | 22.3 ± 19.3 | 23.9 ± 13.4 |
| HsCRP (mg/dL) | 4 ± 3.2 | 4.1 ± 3.5 |
| Values shown are n (%) or means ± standard deviation. * Categories differ significantly from each other at P < 0.05.  HbA1c, Hemoglobin A1c; LDL, Low‐Density Lipoprotein; HDL, High‐Density Lipoprotein; VLDL, Very Low‐Density Lipoprotein; HsCRP, High Sensitivity C-Reactive Protein. | | |

| **Table S3. Changes in Primary And Secondary Endpoints (Long Duration AOM- Non-AOM Group )** | | | | |
| --- | --- | --- | --- | --- |
|  | **Non-AOM**  **(n )** | **Long Duration**  **(n)** | **Difference Between Groups (95% CI)** | **P Value** |
| **Change in weight (%)** |  |  |  |  |
| ITT analysis | 9.99 (628) | 14.34 (321) | -4.4 [5.48, -3.21] | **p<0.01** |
| Digital Completers | 11.21 (421) | 14.75 (284) | -3.5 [-4.91,-2.17] | **p<0.01** |
| Care Completers | 12.77 (235) | 15.66 (240) | -2.9 [-4.42,-1,37] | **p<0.01** |
| **≥ 5% of body weight** |  |  |  |  |
| ITT analysis | 71% | 86.60% | -15.6, [-20.7, -10.5] | **p<0.01** |
| Digital Completers | 75.10% | 87.30% | -12.2, [-17.9, -6.5] | **p<0.01** |
| Care Completers | 83.40% | 90% | -6.6, [-12.7, -0.5] | **0.047** |
| **≥ 10% of body weight** |  |  |  |  |
| ITT analysis | 45.50% | 69.50% | -24.0, [-30.4, -17.6] | **p<0.01** |
| Digital Completers | 52.90% | 71.50% | -18.6, [-25.8, -11.4] | **p<0.01** |
| Care Completers | 63.80% | 76.20% | -12.4, [-20.6, -4.2] | **p<0.01** |
| **≥ 15% of body weight** |  |  |  |  |
| ITT analysis | 24% | 46.40% | -22.4, [-28.8, -16.0] | **p<0.01** |
| Digital Completers | 29.90% | 48.90% | -19.0, [-26.3, -11.7] | **p<0.01** |
| Care Completers | 37.90% | 52.10% | -14.2, [-23.1, -5.3] | **p<0.01** |
| **≥ 20% of body weight (%)** |  |  |  |  |
| ITT analysis | 11% | 26.20% | -15.2, [-20.6, -9.8] | **p<0.01** |
| Digital Completers | 15.20% | 27.50% | -12.3, [-18.6, -6.0] | **p<0.01** |
| Care Completers | 19.10% | 30% | -10.9, [-18.6, -3.2] | **p<0.01** |
| All changes are from baseline to 18 months. Values shown are means ± standard deviation or % of weight reduction target achieved. Bold Categories differ significantly from each other at P < 0.05. | | | | |
|  |  |  |  |  |

| **Table S4. Changes in Primary And Secondary Endpoints (Short Duration AOM- Non-Medication Group )** | | | | |
| --- | --- | --- | --- | --- |
|  | **Non-AOM**  **(n )** | **Short Duration**  **(n)** | **Difference Between Groups (95% CI)** | **P Value** |
| **Change in weight (%)** |  |  |  |  |
| ITT analysis | 9.25 (333) | 9.99 (628) | 0.74(-.035-1.84) | 0.183 |
| Digital Completers | 10.04 (224) | 11.21 (401) | 1.2 (-0.33-2.66) | 0.126 |
| Care Completers | 12.26 (138) | 12.77 (235) | 0.51 (-1.33-2.35) | 0.586 |
| **≥ 5% of body weight** |  |  |  |  |
| ITT analysis | 71.0% | 68.80% | 2.2, [-3.9, 8.3] | 0.51 |
| Digital Completers | 75.10% | 70.10% | 5.0, [-2.3, 12.3] | 0.21 |
| Care Completers | 83.40% | 79.70% | 3.7, [-4.5, 11.9] | 0.45 |
| **≥ 10% of body weight** |  |  |  |  |
| ITT analysis | 45.50% | 40.50% | 5.0, [-1.6, 11.6] | 0.16 |
| Digital Completers | 52.90% | 45.50% | 7.4, [-0.7, 15.5] | 0.09 |
| Care Completers | 63.80% | 58.70% | 5.1, [-5.2, 15.4] | 0.38 |
| **≥ 15% of body weight** |  |  |  |  |
| ITT analysis | 24% | 20.70% | 3.3, [-2.2, 8.8] | 0.28 |
| Digital Completers | 29.90% | 25.40% | 4.5, [-2.8, 11.8] | 0.27 |
| Care Completers | 37.90% | 33.30% | 4.6, [-5.4, 14.6] | 0.44 |
| **≥ 20% of body weight (%)** |  |  |  |  |
| ITT analysis | 11.00% | 11.40% | -0.4, [-4.6, 3.8] | 0.93 |
| Digital Completers | 15.20% | 15.60% | -0.4, [-6.3, 5.5] | 0.98 |
| Care Completers | 19.10% | 21.70% | -2.6, [-11.1, 5.9] | 0.64 |
| All changes are from baseline to 18 months. Values shown are means ± standard deviation or % of weight reduction target achieved. Bold Categories differ significantly from each other at P < 0.05. | | | | |
|  |  |  |  |  |

| **Table S5. Changes in Primary End Points (2nd generation - 1st Generation)** | | | | |
| --- | --- | --- | --- | --- |
|  | **1st Gen AOM users (n)** | **2nd Gen AOM Users**  **(n)** | **Difference Between Groups (95% CI)** | **P Value** |
| **Change in weight (%)** |  |  |  |  |
| ITT analysis | 11.52 (581) | 13.58 (73) | 2.06[-4.23,0.1] | 0.061 |
| Digital Completers | 12.49 (447) | 14.02 (61) | 1.53[-4.02,0.95] | 0.226 |
| Care Completers | 14.28 (327) | 15.33 (51) | 1.05[-3.59,1,49] | 0.416 |
| **≥ 5% of body weight** |  |  |  |  |
| ITT analysis | 77.3 | 79.5 | 2.2[-7.7,12] | 0.787 |
| Digital Completers | 79.9 | 78.7 | -1.2[-12.1,9.8] | 0.964 |
| Care Completers | 86.9 | 82.4 | -4.5[-15.6,6.6] | 0.517 |
| **≥ 10% of body weight** |  |  |  |  |
| ITT analysis | 53.9 | 61.6 | 7.8[-4.1,19.6] | 0.257 |
| Digital Completers | 59.5 | 63.9 | 4.4[-8.5,17.3] | 0.601 |
| Care Completers | 69.4 | 72.6 | 3.1[10.1,16.4] | 0.773 |
| **≥ 15% of body weight** |  |  |  |  |
| ITT analysis | 32.0 | 43.8 | 11.8[-0.2,23.8] | 0.059 |
| Digital Completers | 37.4 | 47.5 | 10.2[-3.1,23.5] | 0.164 |
| Care Completers | 44.0 | 52.9 | 8.9[-5.8,23.6] | 0.300 |
| **≥ 20% of body weight (%)** |  |  |  |  |
| ITT analysis | 17.2 | 30.1 | **12.9[2,23.9]** | **0.012** |
| Digital Completers | 20.8 | 32.8 | 12[-.4,24.3] | 0.052 |
| Care Completers | 25.4 | 37.3 | 11.9[-2.2,26] | 0.108 |
| All changes are from baseline to 18 months. Values shown are means ± standard deviation or % of weight reduction target achieved. Bold Categories differ significantly from each other at P < 0.05. | | | | |

| **Table S6. Changes in Primary End Points (2nd Generation Long - 1st Generation Long)** | | | | |
| --- | --- | --- | --- | --- |
|  | **1st Gen Long AOM users (n)** | **2nd Gen Long AOM Users**  **(n)** | **Difference Between Groups (95% CI)** | **P Value** |
| **Change in weight (%)** |  |  |  |  |
| ITT analysis | 14.01 (272) | 16.16 (49) | 2.15[-4.83,0.53] | 0.116 |
| Digital Completers | 14.48 (240) | 16.21 (44) | 1.73[-4.58,1.12] | 0.234 |
| Care Completers | 15.35(202) | 17.34 (38) | 1.99[-4.8,-0.87] | 0.172 |
| **≥ 5% of body weight** |  |  |  |  |
| ITT analysis | 86.8 | 85.7 | -1.1 [-11.6,9.5] | 1.000 |
| Digital Completers | 87.5 | 86.4 | -1.1[-12.1,9.8] | 1.000 |
| Care Completers | 90.1 | 89.5 | -0.6 [-11.2,10] | 1.000 |
| **≥ 10% of body weight** |  |  |  |  |
| ITT analysis | 68.4 | 75.5 | 7.1[-6.1,20.4] | 0.407 |
| Digital Completers | 70.8 | 75.0 | 4.2[-9.9,18.2] | 0.703 |
| Care Completers | 75.3 | 81.6 | 6.3[-7.4,20] | 0.526 |
| **≥ 15% of body weight** |  |  |  |  |
| ITT analysis | 44.1 | 59.2 | 15.1[0.1,30] | 0.073 |
| Digital Completers | 47.1 | 59.1 | 12[-3.8,27.8] | 0.193 |
| Care Completers | 50.0 | 63.2 | 13.2[-3.7,30] | 0.189 |
| **≥ 20% of body weight (%)** |  |  |  |  |
| ITT analysis | 23.5 | 40.8 | **17.3[2.6,31.9]** | **0.018** |
| Digital Completers | 25.0 | 40.9 | **15.9[0.4,31.4]** | **0.047** |
| Care Completers | 27.2 | 44.7 | **17.5[0.5,34.5]** | **0.049** |
| All changes are from baseline to 18 months. Values shown are means ± standard deviation or % of weight reduction target achieved. Bold Categories differ significantly from each other at P < 0.05. | | | | |
